# 

**Published:** 1853-04

**Authors:** 


VOL. I. NEW SERIES. No. 12.

Medical Times

### THE NORTH-WESTERN

### MEDICAL AND SURGICAL

### JOURNAL:

EDITED BY

W. B. HERRICK, M.D.,

PROFESSOR OF ANATOMY AND PHYSIOLOGY IN RUSH MEDICAL COLLEGE; SURGEON TO THE U. S. MARINE HOSPITAL, ONE OF THE SURGEONS OF THE ILLINOIS GENERAL HOSPITAL, ETC.

ASSISTED BY

H. A. JOHNSON, A.M., M.D.

PUBLISHED MONTHLY,

AT TWO DOLLARS PER ANNUM.

APRIL, 1853.

CHICAGO:

BALLANTYNE & CO., PUBLISHERS & PRINTERS,

NEW POST-OFFICE BUILDINGS, COR. OF CLARK AND RANDOLPH-STS., OPPOSITE THE SHERMAN HOUSE.

1853.

VOL I. WHOLE SERIES. No. 12.

